# Clinical and neuroimaging factors associated with aphasia severity in stroke patients: diffusion tensor imaging study

**DOI:** 10.1038/s41598-020-69741-1

**Published:** 2020-07-30

**Authors:** Sekwang Lee, Yoonhye Na, Woo-Suk Tae, Sung-Bom Pyun

**Affiliations:** 1 Department of Biomedical Sciences, Korea University College of Medicine, Seoul, Korea; 2 Brain Convergence Research Center, Korea University College of Medicine, Seoul, Korea; 3 Department of Physical Medicine and Rehabilitation, Korea University College of Medicine, 73 Goryeodae-ro, Seongbuk-gu, Seoul, 02841 Korea

**Keywords:** Neurological disorders, Stroke

## Abstract

This study investigated factors associated with aphasia severity at both 2 weeks and 3 months after stroke using demographic and clinical variables, brain diffusion tensor imaging (DTI) parameters, and lesion volume measurements. Patients with left hemisphere stroke were assessed at 2 weeks (n = 68) and at 3 months (n = 20) after stroke. Demographic, clinical, and neuroimaging data were collected; language functions were assessed using the Western Aphasia Battery. For neuroimaging, DTI parameters, including the laterality index (LI) of fractional anisotropy (FA), axial diffusivity (AD), radial diffusivity, mean diffusivity and fibre density (FD) of the arcuate fasciculus (AF), and lesion volume, were measured. Lesion volume, cortical involvement, and the National Institutes of Health Stroke Scale score significantly predicted aphasia severity at 2 weeks after stroke, whereas the aphasia quotient and presence of depression during the early subacute stage were significant predictors at 3 months after stroke. According to Pearson correlation, LI-AD and LI-FD were significantly correlated with the aphasia quotient 2 weeks after ischaemic stroke, and the LI-FA was significantly correlated with the aphasia quotient 2 weeks after haemorrhagic stroke, suggesting that the extent and mechanism of AF injuries differ between ischaemic and haemorrhagic strokes. These differences may contribute to aphasia severity.

## Introduction

Aphasia arises from the inability to understand and regulate language after injury to certain areas of the brain^1^. Approximately 35–40% of adults admitted to hospital because of strokes are diagnosed with aphasia^2^. Furthermore, approximately 85% of stroke patients with aphasia recover from the aphasia within 3–6 months after onset; however, some may require additional time for recovery and others may never fully recover^3,4^. Accurately predicting the prognoses of patients with aphasia during the early post-stroke stages can be helpful for determining the appropriate timing of discharge and need for institutional help^5^. Patients with poor post-stroke auditory comprehension and naming ability (anomia) are more often discharged to an institution rather than to home^6^.

Factors associated with recovery from aphasia can be divided into non-lesion- and lesion-related factors^7^. Non-lesion-related factors include age, sex, handedness, and years of education, whereas lesion-related factors include acute-stage aphasia severity, diagnosed aphasia type, and location and size of any corresponding brain lesions^8–10^. Stroke-related variables are robust predictors of recovery; however, patient-related variables have been reported to have minimal or no significant prognostic value^7,9^.

Recently, newly developed techniques, such as diffusion tensor imaging (DTI), voxel-based lesion symptom mapping, and functional magnetic resonance imaging (MRI), have been used to study the effects of brain lesion locations and sizes on aphasia^11–13^. DTI can provide information regarding the degree of damage to and structure of white matter in the brain using the diffusion of water molecules within the tissue to generate images^14^. Values such as fractional anisotropy, axial diffusivity, radial diffusivity, and mean diffusivity can be obtained by analysing DT images, and visualisation of the white matter fibre bundles using analytical software is now possible^15^. Fractional anisotropy has emerged as one of the most useful indices for evaluating subcortical neural structures because it reflects the microstructural integrity^16^.

The arcuate fasciculus (AF) is one of the most important bundles of subcortical neurons associated with language function; it is also a link between the Broca and Wernicke areas of the brain^17^. Several studies have examined the usefulness of DTI for diagnosing aphasia. Investigators have compared the clinical features of aphasia with AF damage; however, to date, few studies have attempted to use DTI to predict aphasia prognoses^18^. DTI studies have reported differences in the asymmetry of fibre numbers in the AF of patients with and without aphasia after ischaemic stroke^19^. Six months after their strokes, patients who did not have a left AF, according to DT tractography, had more severe aphasia than other patient groups^20^. Different stroke types (ischaemic or haemorrhagic) have different functional outcomes, including aphasia^21^. Haemorrhagic stroke patients experience greater disabilities during hospitalisation than ischaemic stroke patients; however, they exhibit greater post-rehabilitation functional recovery^22^. Whether these two stroke types cause different types of damage to the subcortical neural structures remains unclear. Therefore, using DTI to compare the AF details of patients with aphasia, based on stroke type, is necessary.

This study investigated the clinical and neuroimaging factors associated with aphasia severity at both 2 weeks and 3 months after stroke, based on DTI results. Furthermore, differences between these factors were analysed based on the stroke type.

## Methods

### Participants

The Korea University Anam Hospital Stroke Outcome Prediction (STOP) database was developed to predict post-stroke functional recovery, based on demographic, clinical, and neuroimaging data. During this project, the motor, language, and cognitive functions of stroke patients were evaluated during the acute phase (within 1 week), at admission to the rehabilitation ward (approximately 2 weeks), and at 3 and 6 months after stroke. Structural and DTI scans were performed within 4 weeks and at 6 months after stroke onset. This study retrospectively analysed data from the STOP database. The Clinical Research Ethics Committee of Korea University approved the study protocol (no. 2017AN0005), and informed consent was obtained from all individual participants included. All procedures performed during studies involving human participants were in accordance with the ethical standards of the institutional and/or national research committees and with the 1964 Declaration of Helsinki and its later amendments or comparable ethical standards.

Medical records of 470 patients who underwent DTI between July 2013 and November 2017 were reviewed. Patients diagnosed with left middle cerebral artery (MCA) infarctions or thalamic strokes, using brain MRI or computed tomography (CT), were included. Patients with left intracerebral haemorrhage (ICH) were also included. The exclusion criteria were brainstem or cerebellar haemorrhage, which may not affect AF; bilateral stroke lesions; a history of neurological, psychiatric, or speech disorders; and patients who did not undergo an evaluation of language function using a comprehensive language assessment (Korean version of the Western Aphasia Battery, K-WAB) within 4 weeks of stroke onset.

Of the 470 patients, 106 (42 with MCA territory infarctions, 1 with thalamic infarction, 63 with ICHs) were initially included. However, 10 of these 106 patients had cerebellum or brainstem haemorrhages, 3 had bilateral stroke lesions, 14 had histories of stroke or major depression disorder, and 11 had not undergone a language evaluation within 4 weeks of stroke onset. Finally, 68 patients were included in the study; of these, 20 underwent a follow-up language assessment 3 months after their strokes.

### Demographic and clinical data

Medical records were reviewed to collect demographic and clinical variable information, including sex, age, pre-morbid handedness, years of education, Mini-Mental Status Examination (MMSE) results, presence of depressive symptoms, and the initial National Institutes of Health Stroke Scale (NIHSS) scores. Assessments of cognitive function, depressive symptoms, and language were performed 2 weeks after the stroke events. Cognitive function was assessed using the Korean version of the MMSE. The Korean version of Beck Depression Inventory (BDI) or the Korean version of the Geriatric Depression Scale (GDS) was applied to assess depression. When patients were unable to undergo the BDI or GDS assessment because of severe aphasia, their depressive symptoms were carefully observed by a psychiatrist and antidepressants were prescribed accordingly. We organised the results according to the presence or absence of depression (dichotomous) to unify the results obtained in different ways. The following criteria were considered evidence of depressive symptoms: GDS score ≥ 17 (maximum, 30 points), BDI score ≥ 16 (maximum, 63 points), or antidepressant use initiated after clinical assessment^23,24^.

Using brain imaging results, the stroke lesion volumes were determined and the brain lesion locations (cortical/subcortical) were assessed. The brain lesion volumes were measured using CT or T1-weighted MRI performed at the time of the stroke diagnosis. The examiner marked the brain lesion areas on cross-sections of the acquired images using Analyze 9.0 (Biomedical Imaging Resource, Mayo Clinic, Rochester, MN, USA), a semi-automated analysis software program. The program automatically analyses the brain lesion volumes using the segmentation method. Cortical involvement, defined as the presence of signal changes in the cerebral cortex on CT or MRI (including the frontal, parietal, temporal, and occipital lobes), was judged by radiologists. The prognoses for aphasia following subcortical stroke are better than those for strokes with cortical involvement^25^; therefore, the presence or absence of cortical involvement (dichotomous) was used as a variable in the statistical analyses.

Language data for the study were derived from the results of the K-WAB. The core subtests of the K-WAB evaluate spontaneous speech (20 points), comprehension (10 points), repetition (10 points), and naming (10 points); the subscores are combined to calculate the aphasia quotient (AQ). We examined all subtest scores to evaluate the relationships between clinical factors, neuroimaging factors, and language assessment results. The results of the language assessments performed at 2 weeks (AQ1) and 3 months (AQ2) after stroke onset were included. The AQ1 scores the total duration (hours) of speech therapy received were also considered variables in the AQ2 language evaluation and were used for analysis. The total duration of speech therapy was investigated using patient medical records and self-reports of treatments received at other hospitals.

### DTI data acquisition

DTI was performed, using a Siemens Magnetom Prisma 3 T MRI device (Siemens Healthineers, Erlangen, Germany) with a 64-channel head coil, on 68 patients at 4 weeks after stroke onset. Images were acquired based on single-shot echo-planar imaging using 64 directions. The DTI acquisition parameters for the MRI scanner were axial orientation; matrix, 224 × 224; field of view, 224 mm × 224 mm; slice thickness, 2.0 mm; voxel size (*x*, *y*, and *z*), 1.0 × 1.0 × 2.0 (mm); echo time, 55 ms; repetition time, 6,500 ms; *b* = 1,000 s/mm^2^; and flip angle, 90°. In the acquired image, using the mutual information algorithm of the Automatic Image Registration, 64-direction, diffusion-weighted images and 31 b_0_ images were co-registered with the first b_0_ volume acquired^26^.

### Analysis of AF

DTI Studio, version 3.0.2, software (https://ww.mristudio.org; H. Jiang, S, Mori, Department of Radiology, Johns Hopkins University, Baltimore, MD, USA) was used to process and visualise the AF path. Fibre assignment, using continuous tracking reconstruction, was performed to identify the DTI fibre track^15^. The minimum fractional anisotropy (FA) was set at 0.15, and the tract turning angle was set to 70° for tract reconstruction. Two regions of interest (ROIs) were set to delineate the AF. In the coronal plane, at the posterior-most portion of the midline fornix, the periventricular anteroposterior-oriented fibres (green) were set as the first ROI. Periventricular craniocaudally oriented fibres (blue) were set as the second ROI at the axial level of the anterior commissure^27^. The T1-weighted horizontal images were used to clearly identify the anatomic boundary and verify that the ROI settings were correct. A reconstructed three-dimensional fibre was then added to each T1-weighted horizontal image, and the FA, axial diffusivity (AD), radial diffusivity (RD), and mean diffusivity (MD) values and fibre tract density (the number of fibres within the bundle passing through the two ROIs) were extracted. FA and AD are known to decrease when there is microstructural integrity damage and with axonal injury, respectively. RD increases with demyelination and MD increases with decreasing membrane density due to oedema and necrosis. The fibre density (FD) reflects the number of white matter fibres passing through the ROI^28,29^.

Next, a laterality index (LI) was calculated to determine how the indices extracted from the left AF differed from those extracted from the right AF. The LI was calculated by substituting the following equation for the indices obtained from both AFs^30^:

$$ {\text{LI }} = \, \left( {{\text{Left }} - {\text{ Right}}} \right)/\left( {{\text{Left }} + {\text{ Right}}} \right) $$

For instance, when the FA value of the left AF was 0, the LI was − 1, whereas when the FA value on the right AF was 0, the LI was + 1. If values on the left and right sides were equal, then the LI was 0. Therefore, the lower the index of the left AF than the index of the right AF, the closer the LI was to − 1. Five variables (LI-FA, LI-AD, LI-RD, LI-MD, LI-FD) were obtained in this way and used for statistical analyses.

The LIs for patients with non-reconstructed left AFs, due to severe damage, could not be obtained; therefore, we categorised the LI-FA according to its value and defined the non-reconstructed LI-FAs as grade 0. In the reconstructed left AF, the calculated LI-FA was categorised as grade 1, 2, or 3 using the average value of all LI-FAs (Table 4). This grading was used to determine whether patients with non-reconstructed AFs had more severe aphasia.

**Figure 1:**
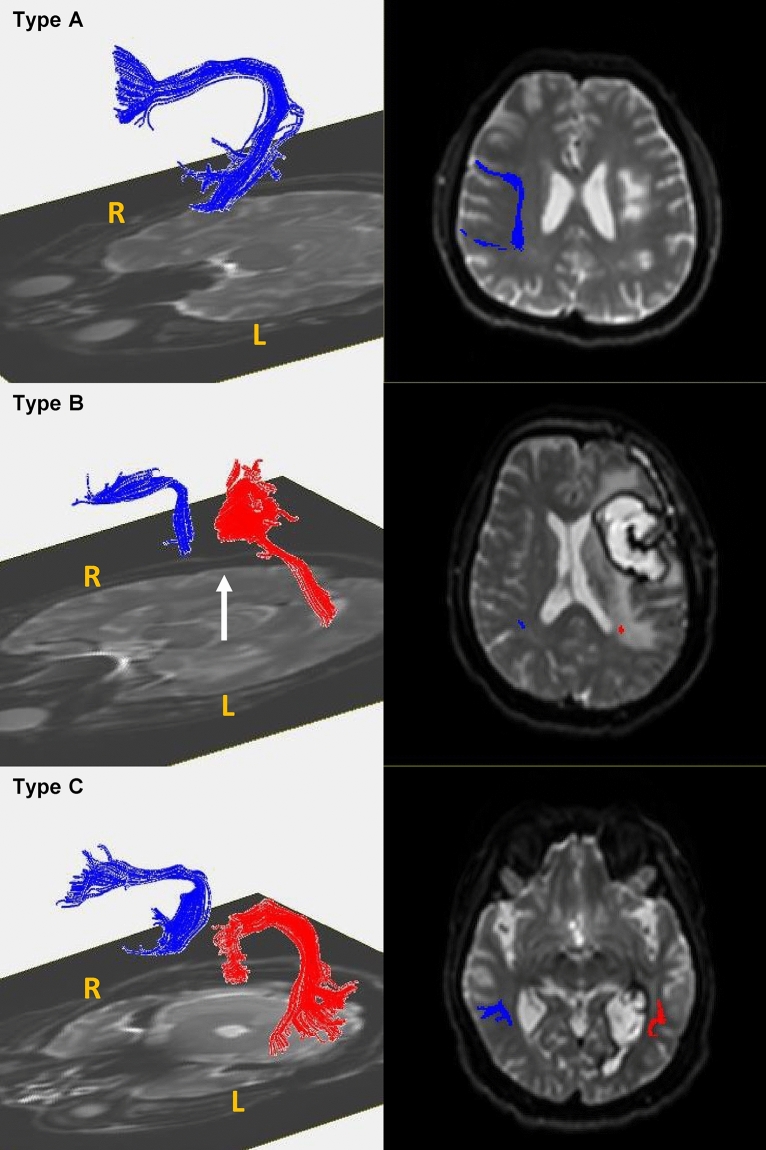
Three types of AF (**A–C**) displayed according to the severity of left AF damage. (1) Diffusion tensor image of the AF and (2) T2-weighted magnetic resonance image showing three types of AF (**A**–**C**; right = blue; left = red) according to the severity of the left AF damage. The white arrow indicates disruption of the left AF surrounding the stroke lesion. *AF* arcuate fasciculus, *R* right, *L* left.

The shape of the AF was analysed and classified into three types (A, B, or C), based on the degree of AF damage^20^. The AF was added to the T1-weighted horizontal image to confirm the anatomical location of the reconstructed AF. Based on this image, the AF was classified as type A when it could not be visualised because of severe damage, type B when the AF damage was observed between the Wernicke and Broca areas, and type C when the AF surrounding the brain lesion was preserved (Fig. 1)^31^.

### Statistical analyses

Comparisons of patient ages, years of education, NIHSS scores, MMSE results, and language assessment scores at both 2 weeks and 3 months after stroke were performed using independent *t*-tests or Mann–Whitney *U*-tests after Shapiro-Wilks tests of normality. Chi-square tests were performed to compare sex distributions, presence of depression, stroke types, and cortical involvement. Independent sample *t*-tests were performed to compare AQ1 and subset scores between the groups with and without cortical involvement. Statistical analyses were performed to determine the factors associated with aphasia severity at 2 weeks and 3 months after stroke.

### Analysis 1

Multiple linear regression, using a stepwise variable selection method, was used to determine the extent that demographic and neurological data contributed to the language assessment result predictions at 2 weeks after stroke. We included seven independent variables—age, sex, years of education, NIHSS score, location (cortical/subcortical), presence of depression, and stroke lesion volume. The language assessment scores, including the subset scores (fluency, comprehension, repetition, and naming) and AQ1, were considered dependent variables. Additionally, a subgroup analysis was performed according to stroke type. Comparisons of clinical characteristics of patients with ischaemic and haemorrhagic strokes were performed using the same methodology used for comparisons between subjects at 2 weeks and 3 months after stroke.

### Analysis 2

A Pearson correlation analysis of the DTI parameters (LI-FA, LI-AD, LI-RD, LI-MD, LI-FD) and language assessment results (AQ1 and subset scores) at 2 weeks after stroke was performed. To determine whether the correlation between the language assessment results and DTI parameters differed according to the stroke type, analyses were performed separately for ischaemic and haemorrhagic stroke patients. Linear associations between all variables were displayed using scatter plots.

### Analysis 3

A multinomial logistic regression analysis was performed to identify the ability of the AQ1 score to predict the LI-FA grade and AF type for 68 stroke patients; the LI-FA grade and AF type were included as dependent variables. An LI-FA grade of 0 (AF not reconstructed) and a type A AF were used as reference categories.

### Analysis 4

To predict language scores at 3 months after stroke, a two-step linear regression analysis was performed. In the simple linear regression to find a comprehensive range of explanatory variables that could be related to dependent variables, multiple testing corrections were not performed. Independent variables with *P* < 0.05 in the simple linear regression analysis were included as independent variables in the multiple linear regression analysis using a stepwise variable selection method. In the simple linear regression, independent variables were demographic and neurological variables used for Analysis 1, DTI parameters used for Analysis 2, AQ1 score, and total duration of speech therapy. The 3-month post-stroke language assessment scores were considered dependent variables. Among these variables, only clinical variables were defined as independent variables for the multiple linear regression analysis (Model 1) to compare the results with those of Analysis 1. Thereafter, we performed a multiple linear regression analysis (Model 2) that included all significant variables from the simple linear regression as independent variables.

All data were analysed using IBM SPSS Statistics, version 24.0 for Windows (IBM, Armonk, NY, USA). In this study, the alpha level was set to 0.05, and *P* < 0.05 was considered statistically significant.

## Results

Sixty-eight patients (36 men and 32 women; mean age, 66.2 years) underwent language evaluations 2 weeks after stroke and were assessed during this study. Twenty patients completed an additional follow-up language assessment 3 months after stroke. The mean AQ1 score was 49.7 ± 31.6 (out of 100), and the mean AQ2 score was 64.8 ± 31.6, which was an improvement of 24.8 ± 20.2 points. Twenty-nine (42.6%) of the 68 patients in the AQ1 group were diagnosed with depressive symptoms. The only significant differences between the AQ1 and AQ2 groups were the higher rates of ischaemic stroke and cortical involvement in the AQ2 group; detailed clinical characteristics are shown in Table 1. The mean language scores were significantly lower for patients with cortical involvement than for those without cortical involvement (Supplementary Table 1 online).

**Table 1:** Clinical characteristics of the study patients.

Variable	2 weeks after onset (n = 68)	3 months after onset (n = 20)	*P* value
Age, years	66.22 ± 11.37	66.24 ± 12.36	0.995
Sex, male/female	36/32	14/6	0.176
Education, years	10.09 ± 5.38	11.90 ± 5.00	0.196
Handedness, right/left	68/0	20/0	
NIHSS score (maximum, 42)	10.46 ± 6.02	10.20 ± 5.83	0.893
MMSE score (maximum, 30)	15.87 ± 9.36	14.75 ± 9.99	0.679
Depression, yes/no	29/39	6/14	0.310
**Yes**	29 (42.6)	6 (30)	
GDS score ≥17 (maximum, 30)	5	0	
BDI score ≥16 (maximum, 63)	10	3	
Clinical assessment	14	3	
**No**	39 (57.4)	14 (70)	
GDS score <17 (maximum, 30)	5	1	
BDI score <16 (maximum, 63)	13	5	
Clinical assessment	21	8	
**Type of stroke, n (%)**			0.048*
**Ischaemic**	34 (50)	15 (75)	
MCA territory	33 (97.1)	15 (100)	
Thalamic	1 (2.9)	0 (0)	
**Haemorrhagic**	34 (50)	5 (25)	
Lobar ICH	10 (29.4)	2 (40)	
Deep ICH	24 (70.6)	3 (60)	
Cortical involvement, yes/no	37/31	16/4	0.040*
Brain volume (cm³)	1420.88 ± 120.27	1474.45 ± 118.80	0.085
Lesion volume (cm³)	39.53 ± 76.61	62.61 ± 121.56	0.308
Interval from stroke to K-WAB (weeks)	2.14 ± 0.97	11.68 ± 0.25	<0.001**
**K-WAB score (maximum)**			
Fluency (20)	10.2 ± 6.6	13.1 ± 6.1	0.013*
Comprehension (10)	5.0 ± 3.0	6.9 ± 3.1	<0.001**
Repetition (10)	5.2 ± 3.8	6.2 ± 3.7	<0.001**
Naming (10)	4.5 ± 3.6	6.2 ± 3.6	<0.001**
AQ (100)	49.7 ± 31.6	64.8 ± 31.6	0.031*
△K-WAB score	–	24.8 ± 20.2	
Total duration of speech therapy (hours)	–	16.6 ± 9.5	

Notes: Values are presented as means ± standard deviation or numbers.

Years of education and the NIHSS, MMSE, and K-WAB scores were compared using the independent t-test. Age was compared using the Mann-Whitney U-test. Sex distribution, presence of depression, stroke type, and cortical involvement were compared using the chi-square test.

*MCA* middle cerebral artery, *ICH* intracerebral haemorrhage, *NIHSS* National Institutes of Health Stroke Scale, *MMSE* Mini-Mental Status Examination, *K-WAB* Korean version of the Western Aphasia Battery, *AQ* aphasia quotient, *GDS* Geriatric depression scale, BDI, Beck Depression Inventory.

*P <0.05, **P <0.01.

Thirty-four patients presented with cerebral infarctions, and an equal number presented with cerebral haemorrhage. Among the 34 patients with cerebral infarctions, 33 were diagnosed with MCA territory infarctions and 1 with a thalamic infarction. Of the 34 patients with cerebral haemorrhages, 10 were diagnosed with lobar haemorrhage and 24 with deep ICH. A comparison of the stroke types indicated that the ischaemic stroke group had more cortical involvement than the haemorrhagic group. The other clinical variables were similar between the groups (Supplementary Table 2 online).

**Table 2:** Results of the multiple linear regression analysis, using stepwise variable selection at 2 weeks after stroke (n = 68).

Dependent variables	Independent variables	Standardised *β*	Adjusted *R*^2^	*P* value	VIF
Fluency			0.564		
	NIHSS	− 0.654		< 0.001**	1.014
	Lesion volume	− 0.318		< 0.001**	1.014
Comprehension			0.321		
	NIHSS	− 0.557		< 0.001**	1.005
	Age	− 0.219		0.034*	1.005
Repetition			0.370		
	NIHSS	− 0.424		< 0.001**	1.022
	Lesion volume	− 0.255		0.015*	1.109
	Cortical involvement	− 0.253		0.016*	1.110
Naming			0.476		
	NIHSS	− 0.590		< 0.001**	1.014
	Lesion volume	− 0.316		0.001*	1.014
AQ			0.555		
	NIHSS	− 0.627		< 0.001**	1.022
	Lesion volume	− 0.239		0.007**	1.109
	Cortical involvement	− 0.187		0.033*	1.110

Independent variables: age, sex, years of education, NIHSS score, location (cortical/subcortical), presence or absence of depression, and stroke lesion volume

*VIF* variance inflation factor, *AQ* aphasia quotient, *NIHSS* National Institutes of Health Stroke Scale

### Analysis 1. Factors associated with aphasia severity at 2 weeks after stroke

Multiple linear regression analysis performed to predict the AQ1 for all patients identified a significant regression equation with an adjusted *R*^2^ of 0.555 (*F*_(3,64)_ = 28.891; *P* < 0.001) using the NIHSS score, lesion volume, and cortical involvement (Table 2). The results of our analysis revealed that the NIHSS score was not only a significant predictor of AQ1 but also a significant variable for all language assessment subset scores. Lesion size was a significant predictor of fluency, repetition, and naming scores, with negative beta coefficients. Age was a significant variable for predicting comprehension, with negative beta coefficients; cortical involvement was a significant predictor of repetition scores, with negative beta coefficients.

According to the subgroup analysis of ischaemic strokes, based on the multiple linear regression analysis results, we identified a significant regression equation with an adjusted *R*^2^ of 0.603 (*F*_(2,31)_ = 26.108; *P* < 0.001) using the NIHSS score and lesion volume (*P* < 0.001) (Supplementary Table 3 online). The results showed that the NIHSS score was a significant predictor of AQ1 and all language assessment subset scores, with negative beta coefficients. Lesion size was a significant predictor of fluency, repetition, and naming scores, with negative beta coefficients.

**Table 3:** Neuroanatomical characteristics of the patients.

Variable	2 weeks after onset (n = 68)	3 months after onset (n = 20)
Time interval from stroke to DTI (days)	26.65 ± 12.99	28.30 ± 16.02
**AF type, n (%)**		
A	15 (22.1)	4 (20)
B	30 (44.1)	9 (45)
C	23 (33.8)	7 (35)
**DTI parameters**		
LI-FA*	− 0.0337 ± 0.0549	− 0.0418 ± 0.0537
LI-AD*	0.0083 ± 0.0307	0.0063 ± 0.0210
LI-RD*	0.0413 ± 0.058 2	0.0427 ± 0.0448
LI-MD*	0.0246 ± 0.0386	0.0243 ± 0.0249
LI-FD*	− 0.1489 ± 0.6329	− 0.0967 ± 0.6497

Values are presented as means ± standard deviation or numbers.

*DTI* diffusion tensor imaging, *AF* arcuate fasciculus, *LI* laterality index, *FA* fractional anisotropy, *AD* axial diffusivity, *RD* radial diffusivity, *MD* mean diffusivity, *FD*, fibre density.

*The mean laterality index values for FA, AD, RD, and MD were calculated from the data of 53 patients during the early subacute stage and from the data of 16 patients during the late subacute stage when AF visualisations were available.

Multiple linear regression analysis of haemorrhagic strokes identified a significant regression equation with an adjusted *R*^2^ of 0.427 (*F* (2,31) = 13.287; *P* < 0.001) using the NIHSS score and lesion volume (*P* < 0.001) (Supplementary Table 4 online). The results revealed that the NIHSS score was a significant predictor of AQ1 and all language assessment subset scores, with negative beta coefficients. Lesion size was a significant predictor of fluency, repetition, and naming scores; cortical involvement was a significant predictor of the comprehension score, with negative beta coefficients.

**Table 4:** Graded laterality index (LI) of fraction anisotropy (FA) and aphasia quotient (AQ) score at 2 weeks after stroke.

Grade	Description	AQ	
		Ischaemic stroke (n = 34)	Haemorrhagic stroke (n = 34)
0	AF was not reconstructed	17.4 ± 9.5 (8)	37.7 ± 9.9 (7)
1	LI-FA < − 0.030	51.6 ± 7.0 (16)	38.1 ± 8.1 (9)
2	− 0.030 ≤ LI-FA < 0	54.6 ± 13.7 (5)	78.8 ± 8.0 (7)
3	LI-FA ≥ 0	67.7 ± 17.7 (5)	58.6 ± 9.2 (11)

The median LI-FA value was − 0.030.

*AF* arcuate fasciculus, *LI-FA* laterality index of fraction anisotropy.

### Analysis 2. DTI parameters and aphasia severity at 2 weeks after stroke

On average, DTI was performed approximately 26.65 ± 12.99 days after stroke. Classifications based on the AF shape indicated that type B was the most common (30 cases), followed by types C (23 cases) and A (15 cases; Table 3). Patients with grade 0 LI-FAs had the lowest AQ1, and the AQ1 increased from grade 1 to grade 3 for patients experiencing ischaemic strokes. However, the AQ did not increase with the LI-FA grade for patients experiencing haemorrhagic strokes (Table 4).

The Pearson correlation analysis of the relationship between the DTI parameters and language assessment results at 2 weeks after stroke revealed that LI-AD and LI-FD were positively correlated with AQ1, indicating better language function at higher left-side brain ADs or FDs in patients experiencing ischaemic stroke. In particular, the LI-FD was positively correlated with fluency, comprehension, and naming scores, and LI-AD was positively correlated with fluency and naming scores. The LI-FA was not correlated with the AQ score; however, it was positively correlated with the repetition and naming scores. The analysis of haemorrhagic strokes indicated that the LI-FA was positively correlated with AQ1, fluency, and naming scores. The LI-FA was the only variable positively correlated with naming scores in both the ischaemic and haemorrhagic stroke groups (Table 5, Supplementary Fig. 1 online).

**Table 5:** Results of the Pearson correlation analysis at 2 weeks after stroke.

Variables		Fluency		Comprehension		Repetition		Naming		AQ score	
		Pearson correlation	*P* value	Pearson correlation	*P* value	Pearson correlation	*P* value	Pearson correlation	*P* value	Pearson correlation	*P* value
Ischaemic (n = 26)	LI-FA	0.259	0.201	0.273	0.178	0.459*	0.018	0.412*	0.036	0.378	0.057
	LI-AD	0.460*	0.018	0.358	0.072	0.259	0.201	0.511**	0.008	0.456*	0.019
	LI-RD	0.098	0.632	− 0.032	0.876	− 0.245	0.227	− 0.058	0.780	− 0.038	0.854
	LI-MD	0.337	0.092	0.167	0.416	− 0.045	0.825	0.244	0.229	0.227	0.266
	LI-FD^a^	0.411*	0.016	0.468**	0.005	0.235	0.181	0.364*	0.034	0.403*	0.018
Haemorrhagic (n = 27)	LI-FA	0.456*	0.017	0.277	0.161	0.310	0.115	0.496**	0.009	0.405*	0.036
	LI-AD	− 0.026	0.897	− 0.002	0.994	0.020	0.923	0.101	0.616	0.036	0.858
	LI-RD	− 0.413*	0.032	− 0.249	0.210	− 0.258	0.194	− 0.368	0.059	− 0.335	0.088
	LI-MD	− 0.315	0.109	− 0.186	0.353	− 0.183	0.360	− 0.238	0.232	− 0.236	0.237
	LI-FD^a^	0.330	0.057	0.131	0.461	0.290	0.096	0.338	0.051	0.288	0.098

Variables that demonstrate statistically significant correlations with AQ scores are shown in bold.

The LI-FD was calculated using data from each of the 34 patients with ischaemic and haemorrhagic strokes.

*AQ* aphasia quotient, *LI* laterality index, *FA* fraction anisotropy, *AD* axial diffusivity, *RD* radial diffusivity, *MD* mean diffusivity, *FD* fibre density.

*P < 0.05, **P < 0.01.

^a^LI-FD was calculated from the data of 34 ischaemic and 34 haemorrhagic stroke patients.

### Analysis 3. Relationship between AF damage and language scores

Multinomial logistic regression analysis found a statistically significant relationship between the AQ and LI-FA grade. The AQ had a statistically significant role in differentiating LI-FA grades 1, 2, and 3 from LI-FA grade 0 and AF types B and C from type A (Table 6).

**Table 6:** Estimated effects of the aphasia quotient score at 2 weeks after stroke in the multinomial logistic regression analysis of the laterality index of fractional anisotropy (LI-FA) grade or arcuate fasciculus (AF) type.

Dependent variables	*P* value			Exp (β)	95% CI			
					Lower		Upper	
**LI-FA grade**								
1	0.051			1.024	1.000		1.048	
2	0.002**			1.053	1.020		1.088	
3	0.004**			1.042	1.013		1.071	
0^a^				1				
**AF type**								
B	0.022*			1.027	1.004		1.051	
C	0.001**			1.045	1.018		1.072	
A^a^				1				

The value of the aphasia quotient score in the logistic regression test was *P* = 0.001

*CI* confidence interval

*P < 0.05, **P < 0.01.

^a^Reference category

### Analysis 4. Factors associated with aphasia severity at 3 months after stroke

A two-step linear regression analysis was performed. In the simple linear regression analysis, AQ1 was a significant predictor, with positive beta coefficients, and depression was also a significant predictor, with negative beta coefficients, of AQ2 scores and all subset scores. Furthermore, the NIHSS score was a significant predictor of fluency, with negative beta coefficients (Supplementary Table 5 online). These three statistically significant variables (AQ1, presence of depression, NIHSS score) in the simple linear regression analysis were included as independent variables in the multiple regression analysis.

The presence of depression and NIHSS score (clinical variables) were selected as independent variables for the multiple linear regression Model 1 analysis. We identified a regression equation with an adjusted *R*^2^ of 0.281 (*F*_(1,18)_ = 8.410; *P* < 0.010) to predict the AQ2 in Model 1. All significant variables (AQ1, presence of depression, NIHSS score) in the simple linear regression were included as independent variables in Model 2 of the multiple linear regression analysis. We identified a significant regression equation with an adjusted *R*^2^ of 0.680 (*F*_(2,17)_ = 21.141; *P* < 0.001) to predict AQ2 (Table 7). The presence of depression remained a significant predictor of AQ2, with negative beta coefficients, even when AQ1 was included in the regression analysis as an independent variable. Additional multiple linear regression analyses based on the stroke type were not conducted because of the small sample size.

**Table 7:** Results of the multiple linear regression analysis, using stepwise variable selection at 3 months after stroke (n = 20).

Model	Dependent variables	Independent variables	Standardised *β*	Adjusted *R*^2^	*P* value	VIF
Model 1	Fluency			0.348		
		Depression	− 0.457		0.026*	1.017
		NIHSS	− 0.401		0.047*	1.017
	Comprehension			0.281		
		Depression	− 0.565		0.009**	1.000
	Repetition			0.270		
		Depression	− 0.555		0.011*	1.000
	Naming			0.240		
		Depression	− 0.529		0.016*	1.000
	AQ2			0.281		
		Depression	− 0.564		0.010**	1.000
Model 2	Fluency			0.548		
		AQ1	0.756		< 0.001**	1.000
	Comprehension			0.478		
		AQ1	0.490		0.013*	1.120
		Depression	− 0.404		0.034*	1.120
	Repetition			0.607		
		AQ1	0.618		0.001**	1.120
		Depression	− 0.353		0.033*	1.120
	Naming			0.678		
		AQ1	0.695		< 0.001**	1.120
		Depression	− 0.301		0.043*	1.120
	AQ2			0.680		
		AQ1	0.665		< 0.001**	1.120
		Depression	− 0.346		0.022*	1.120

Statistically significant variables in the simple linear regression analysis were included as independent variables in the multiple linear regression analysis.

Model 1: Presence of depression and NIHSS score were independent variables.

Model 2: Presence of depression, NIHSS score, and AQ1 were independent variables.

*NIHSS* National Institutes of Health Stroke Scale, *AQ* aphasia quotient, *AQ1* aphasia quotient at 2 weeks after stroke onset, *AQ2* aphasia quotient at 3 months after stroke onset, *VIF* variance inflation factor.

*P < 0.05, **P < 0.01.

## Discussion

This study investigated factors associated with aphasia severity at both 2 weeks and 3 months after stroke. The results of our study showed that aphasia severity at 2 weeks was significantly associated with stroke severity (the NIHSS score), lesion volume, and the presence of cortical involvement. Among the DTI parameters, the LI-AD and LI-FD were significantly correlated with the AQ during the early subacute stage of ischaemic strokes, whereas the LI-FA demonstrated a significant correlation in patients with haemorrhagic stroke. However, no significant association was observed between the DTI parameters and aphasia severity at 3 months after stroke. AQ1 was the strongest predictor of AQ2; the presence of depression was also a significant predictor.

FA is one of the most commonly used DTI parameters^18^. DTI is useful for evaluating AF damage severity and for classifying aphasia^32^. Another study reported the association of aphasia severity with the LI-FD, but not with the LI-FA, in MCA infarctions^19^. Consistent with these previous studies, we showed that the LI-FD and LI-AD were significantly correlated with AQ1 in ischaemic strokes; however, only the LI-FA showed a significant correlation with the AQ1 scores in haemorrhagic strokes. These findings suggest that the extent and mechanism of AF injury differ between ischaemic and haemorrhagic strokes, and these differences may contribute to the initial aphasia severity and extent of language function recovery during the subacute phase^21,22^. Basso et al*.*^33^ reported that aphasia associated with haemorrhagic stroke has a better prognosis than that associated with ischaemic stroke because the haemorrhage may cause dislocation of the AF fibre bundles but does not completely destroy them, as occurs in patients experiencing ischaemic stroke. This finding was supported by a recent study using DT tractography that showed displacement of the subcortical neural structures due to compression effects of the hematoma in intracerebral haemorrhage^34^.

The lesion volume was a significant variable for predicting AQ1 scores. As the size of the brain lesions increased, the AQ1 tended to decrease; this tendency was the same across all K-WAB subset scores, except the comprehension score. After categorisation of the brain regions, Maas et al*.*^35^ reported that patients with more involved brain regions had poorer recovery from aphasia than those with fewer involved areas. Additionally, in a study using voxel-based lesion symptom mapping, Henseler et al*.*^36^ suggested that the aphasia type could be classified according to the brain lesion location. Another study reported that the AQ scores were higher in patients with subcortical lesions, followed by in those with cortical lesions involving the Broca and Wernicke areas, and were the least in patients with insula and cortical lesions not in the Broca or Wernicke areas^25^. In our study, patients in the subcortical group displayed higher mean AQ scores than those in the cortical group 2 weeks after stroke (Supplementary Table 1 online), but the specific stroke lesion locations were not included in the study. To determine the effects of lesion location and size, a new approach to predicting aphasia severity using the AF lesion load (AF-LL) has been introduced. This concept is used to calculate the overlapping region of the AF in stroke patients with the canonical AF tract in healthy controls. Marchina et al*.*^37^ reported that the AF-LL can predict speech impairment. Wang et al*.*^38^ also reported that using the AF-LL method, speech fluency and naming ability can be predicted, with a high accuracy (≥ 90%), for patients in the severe and non-severe outcome groups. In our study, the presence of cortical involvement was used in the analysis instead of detailed information regarding lesion locations. The accuracy of aphasia prediction will likely improve by combining lesion site and size as variables, such as in the AF-LL analysis.

The multinomial logistic regression analysis revealed that AQ1 differentiated the LI-FA grades and that the AF types classified the AF shapes using qualitative methods. Recently, Kim et al*.*^20^ reported that the AQ was significantly higher for patients classified as type C than for those classified as type A; however, although they found a significant difference in the AQ based on the AF classification after 6 months, they did not find a significant difference in the AQ of patients classified as type A and those classified as type B. Based on the results of our study, we believe that the AF classification determined using the qualitative and quantitative approaches is significantly related to language function during the early subacute phase of stroke recovery.

In our study, AQ1 and depression were significant predictors of AQ2. Lazar et al*.*^10^ also reported that the initial AQ during the post-stroke acute phase was predictive of recovery from aphasia after 3 months. In agreement with their study, we found that aphasia severity during the early post-stroke phase was a statistically significant and strong variable for predicting recovery at 3 months. Some reports have indicated that speech therapy has a positive effect on recovery from aphasia^39^. However, the correlation between the total duration of speech therapy and AQ2 was not confirmed in this study. Although the presence of depression was not a significant predictor of aphasia severity at 2 weeks after stroke, it (along with AQ1) was a significant predictor of AQ2. Morris et al*.*^40^ reported that depressive symptoms inhibit post-stroke recovery of functional status, cognitive function, and daily activities. In our study, approximately half of the patients could not undergo questionnaire-based depression screening due to severe aphasia. However, because these patients were observed by a psychiatrist and were prescribed medications, accordingly, we were able to assess the presence of depression through medical records that indicated post-stroke antidepressant use.

Post-stroke aphasia is known to be restored by interactions between factors related to brain lesions, treatment factors, and patient characteristics^7^. In our study, the NIHSS score, lesion size, and lesion-related DTI parameters were significant factors influencing the AQ during the early subacute stage. Unfortunately, these variables were not predictive of the late subacute-stage AQ; rather, depression appeared to be a significant predictor of AQ2. This suggests that not only careful evaluation of the brain lesion status but also consideration of patient-related factors, such as depression, are essential for establishing a treatment strategy for post-stroke aphasia and for predicting patient prognosis.

This study had several limitations. First, the sample size and possible bias in this study must be considered; only 20 patients were included to predict AQ2; whether the follow-up of this small group represents the 68 patients participating in the initial evaluation is unknown. A between-group comparison indicated that the ischaemic stroke and cortical involvement ratios were higher for the follow-up group than for the AQ1 group. Because ischaemic stroke and cortical involvement are associated with worse and more persistent aphasia^25,41^, the follow-up group may have had more severe aphasia than the AQ1 group. Second, multiple testing correction was not performed during the multiple univariate regression analysis in “Analysis 4”. Although the purpose of our research was to find a wide range of explanatory factors related to aphasia severity, the absence of this type of correction may have led to an inflated critical *P-*value in the statistical analysis. Third, several white matter tracts have been reported to be related to language function, but only the AF was used in the present analysis. Historically, the AF has been considered the main tract associated with language processing; however, other tracts, such as the superior/inferior longitudinal fasciculus, inferior frontal occipital fasciculus, and uncinate fasciculus, are also involved in language function^42^. Therefore, the use of only the AF during the aphasia analysis may have underestimated the DTI technique’s usefulness for predicting aphasia severity. In future, analysing aphasia severity using the multiple white matter tracts involved in language function will be necessary. Finally, although we selected patients with aphasia from a prospective database (STOP), the timings of language assessments and DTIs were inconsistent. Therefore, the absence of a link between the late phase of recovery and neuroimaging variables should be cautiously interpreted.

Despite these limitations, the authors believe that this study provides important information about clinical and neuroimaging predictors of aphasia severity during the early and late subacute phases of strokes. Our subgroup analysis, based on ischaemic and haemorrhagic strokes, provides an understanding of differences in the mechanism of AF damage after brain insults. This information will be useful for predicting recovery from aphasia, according to the different post-stroke phases, and for creating rehabilitation strategies for patients with aphasia. Future studies using the multiple white matter tracts related to language function and long-term follow-up with larger sample sizes will increase the ability to predict aphasia severity.

## Conclusion

This study aimed to identify the role of neuroimaging parameters, including lesion volume and AF from DTI, associated with aphasia severity during the early and late subacute phases of stroke. The results suggest that the lesion volume significantly predicts aphasia severity at 2 weeks after stroke, as do cortical involvement and the NIHSS score. The AQ and presence of depression during the early subacute stage were significant predictors of aphasia severity at 3 months after stroke. According to the Pearson correlation analysis, the LI-AD and LI-FD were significantly correlated with the AQ at 2 weeks after stroke for ischaemic stroke and with the LI-FA for haemorrhagic stroke. These findings suggest that the extent and mechanism of AF injury differ between patients experiencing ischaemic and haemorrhagic strokes and that these differences contribute to aphasia severity.

## Supplementary information

Supplementary Information.

## Data Availability

The datasets generated and/or analysed during the current study are available from the corresponding author upon reasonable request.
