# Supplementary material for: Clinical and neuroimaging factors associated with aphasia severity in stroke patients: diffusion tensor imaging study
Source: Sci Rep. 2020 Jul 30;10:12874. doi: 10.1038/s41598-020-69741-1 (PMC7393375; doi:10.1038/s41598-020-69741-1)
Supplement: Supplementary file 1 — Supplementary Information. [file 41598_2020_69741_MOESM1_ESM.pdf]

Supplementary information

Title: Clinical and Neuroimaging Factors Associated with Aphasia Severity in Stroke Patients: Diffusion Tensor Imaging Study

Authors: Sekwang Lee[1], Yoonhye Na[1], Woo-Suk Tae[2], Sung-Bom Pyun[2,3]*

Affiliations:
[1] Department of Biomedical Sciences, Korea University College of Medicine, Seoul, Korea
[2] Brain Convergence Research Center, Korea University College of Medicine, Seoul, Korea
[3] Department of Physical Medicine and Rehabilitation, Korea University College of Medicine, Seoul, Korea

Corresponding Author Information:
Sung-Bom Pyun, MD, PhD
Department of Physical Medicine and Rehabilitation, Korea University Anam Hospital, Korea University College of Medicine, 73 Goryeodae-ro, Seongbuk-gu, Seoul 02841, Korea
Tel: +82-2-920-6483, Fax: +82-2-929-9951, E-mail: rmpyun@korea.ac.kr

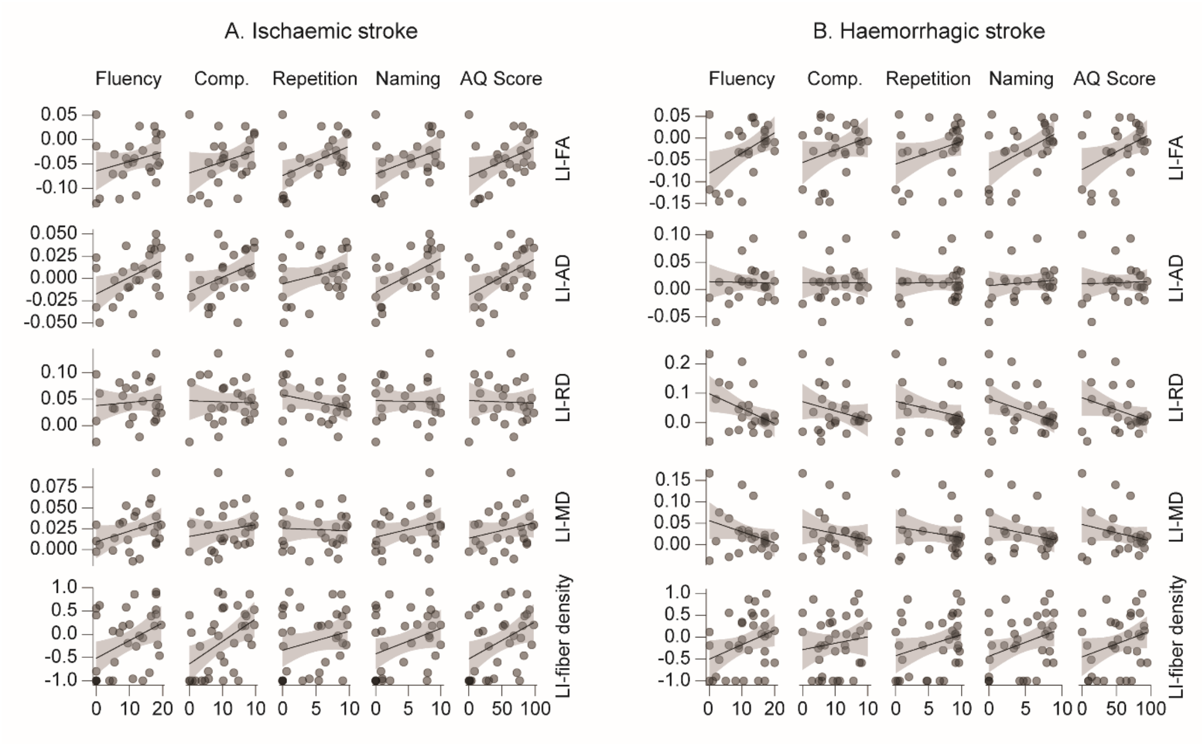

Supplementary Fig. 1 Scatterplot of the Pearson correlation analysis at 2 weeks post-stroke.

Fluency, comprehension (Comp.), repetition, naming scores, and aphasia quotient (AQ) scores of the language evaluation at 2 weeks post-stroke are plotted on the x-axis. LI-FA, LI-AD, LI-RD, LI-MD, and LI fibre density are plotted on the y-axis.

Abbreviations: LI, laterality index; FA, fraction anisotropy; AD, axial diffusivity; RD, radial diffusivity; MD, mean diffusivity.

Supplementary Tables

Supplementary Table 1. Results of independent sample *t*-tests of language assessments conducted at 2 weeks after strokefor the groups with and without cortical involvement.

Language domain	Mean ± SD		*t*	*P* value
	Non-cortical involvement	Cortical involvement		
Fluency	12.5 ± 5.6	8.3 ± 6.9	2.69	0.009**
Comprehension	5.9 ± 2.8	4.3 ± 2.9	2.23	0.029*
Repetition	6.8 ± 3.4	3.9 ± 3.7	3.34	0.001**
Naming	5.7 ± 3.3	3.6 ± 3.4	2.52	0.014*
AQ	61.2 ± 28.7	40.1 ± 31.0	2.88	0.005**

*$P < 0.05$, **$P < 0.01$.

AQ, aphasia quotient; SD, standard of deviation.

Supplementary Table 2. Clinical characteristics of patients with ischaemic and haemorrhagic stroke at 2 weeks after stroke

Variable	Ischemic stroke (n = 34)	Hemorrhagic stroke (n = 34)	*P* value
Age, years	68.72 ± 2.09	63.72 ± 1.72	0.069
Sex, male/female	20/14	16/18	0.331
Education, years	10.56 ± 0.91	9.62 ± 0.94	0.492
Handedness, right/left	34/0 (100)	34 /0 (100)	
NIHSS score (maximum, 42)	10.29 ± 1.11	10.62 ± 0.96	0.827
MMSE score (maximum, 30)	16.5 ± 1.71	15.24 ± 1.51	0.581
Depression, yes/no	14/20	15/19	0.806
Cortical involvement, yes/no	27/7	10/24	< 0.001**
Brain volume (cm³)	1407.44 ± 130.59	1434.32 ± 111.50	0.365
Lesion volume (cm³)	37.94 ± 69.66	16.85 ± 23.41	0.102
Interval between stroke and K-WAB (weeks)	1.96 ± 0.18	2.32 ± 0.15	0.134
K-WAB score (maximum)			
Fluency (20)	9.4 ± 1.2	11.1 ± 1.1	0.386
Comprehension (10)	4.9 ± 0.5	5.1 ± 0.5	0.883
Repetition (10)	4.5 ± 0.7	5.9 ± 0.6	0.058
Naming (10)	4.4 ± 0.7	4.7 ± 0.6	1.000
AQ (100)	46.4 ± 5.7	53.0 ± 5.1	0.440

Notes: Values are presented as means ± standard deviations or number.
Years of education, NIHSS, MMSE, and K-WAB scores were compared using the Mann-Whitney *U*-test. Ages were compared using the independent *t*-test. Sex distribution, presence of depression, and cortical involvement were compared using chi-square tests.

MCA, middle cerebral artery; ICH, intracerebral hemorrhage; NIHSS, National Institutes of Health Stroke Scale; MMSE, Mini-Mental Status Examination; K-WAB, Korean version of the Western Aphasia Battery; AQ, aphasia quotient.

Supplementary Table 3. Results of the multiple linear regression analysis, using stepwise variable selection, at 2 weeks after ischaemic stroke (n = 34).

Dependent variables	Independent variables	Standardized *β*	Adjusted R^2	*P* value	VIF
Fluency			0.654		
	NIHSS	−0.701		<0.001**	1.029
	Lesion volume	−0.328		0.004**	1.029
Comprehension			0.228		
	NIHSS	−0.501		0.003**	1.000
Repetition			0.423		
	NIHSS	−0.544		<0.001**	1.029
	Lesion volume	−0.322		0.023*	1.029
Naming			0.550		
	NIHSS	−0.632		<0.001**	1.029
	Lesion volume	−0.330		0.009**	1.029
AQ			0.603		
	NIHSS	−0.683		<0.001**	1.029
	Lesion volume	−0.304		0.010*	1.029

* $P < 0.05$, ** $P < 0.01$.

AQ, aphasia quotient; NIHSS, National Institutes of Health Stroke Scale; VIF, variance inflation factor.

Supplementary Table 4. Results of multiple linear regression analysis, using stepwise variable selection, at 2 weeks after haemorrhagic stroke (n = 34).

Dependent variables	Independent variables	Standardized β	Adjusted R^2	*P* value	VIF
Fluency			0.436		
	NIHSS	−0.621		0.001**	1.001
	Lesion volume	−0.276		0.043*	1.001
Comprehension			0.422		
	NIHSS	−0.617		<0.001**	1.004
	Cortical involvement	−0.317		0.023*	1.004
Repetition			0.206		
	NIHSS	−0.373		0.022*	1.001
	Lesion volume	−0.331		0.041*	1.001
Naming			0.365		
	NIHSS	−0.542		<0.001**	1.001
	Lesion volume	−0.320		0.028*	1.001
AQ			0.427		
	NIHSS	−0.614		<0.001**	1.001
	Lesion volume	−0.278		0.043*	1.001

*$P < 0.05$, **$P < 0.01$.

AQ, aphasia quotient; NIHSS, National Institutes of Health Stroke Scale; VIF, variance inflation factor.

Supplementary Table 5. Results of the univariate linear regression analysis at 3 months after stroke (n = 20).

Dependent variables	Independent variables	Standardised *β*	Adjusted R^2	*P* value
Fluency	NIHSS	−0.460	0.168	0.041*
	Depression	−0.509	0.218	0.022*
	AQ1	0.756	0.548	<0.001**
Comprehension	Depression	−0.565	0.281	0.009**
	AQ1	0.622	0.353	<0.003**
Repetition	Depression	−0.555	0.270	0.011*
	AQ1	0.733	0.512	<0.001**
Naming	Depression	−0.529	0.240	0.016*
	AQ1	0.794	0.610	<0.001**
AQ2	Depression	-0.564	0.281	0.010*
	AQ1	0.779	0.584	<0.001**

*P <0.05. **P <0.01.

Independent variables: age, sex, years of education, NIHSS score, location (cortical/subcortical), presence/absence of depression, and stroke lesion volume.

AQ, aphasia quotient; AQ1, aphasia quotient at 2 weeks after stroke onset; AQ2, aphasia quotient at 3 months after stroke onset; NIHSS, National Institutes of Health Stroke Scale.
